# Citrate anion improves chronic dialysis efficacy, reduces systemic inflammation and prevents Chemerin-mediated microvascular injury

**DOI:** 10.1038/s41598-019-47040-8

**Published:** 2019-07-23

**Authors:** Sergio Dellepiane, Davide Medica, Cesare Guarena, Tiziana Musso, Alessandro Domenico Quercia, Gianluca Leonardi, Marita Marengo, Massimiliano Migliori, Vincenzo Panichi, Luigi Biancone, Francesco Pizzarelli, Giovanni Camussi, Vincenzo Cantaluppi

**Affiliations:** 1 Nephrology, Dialysis and Kidney Transplantation Unit, Department of Medical Sciences, University of Torino, “Città della Salute e della Scienza” University Hospital, Torino, Italy; 2 Microbiology and Virology Unit, Department of Pathology, University of Torino, “Città della Salute e della Scienza” University Hospital, Torino, Italy; 3 Nephrology and Kidney Transplantation Unit, Department of Translational Medicine and Center for Autoimmune and Allergic Diseases (CAAD), University of Piemonte Orientale (UPO), “Maggiore della Carità” University Hospital, Novara, Italy; 4 Nephrology and Dialysis Unit, ASLCN1, Cuneo, Italy; 5 Nephrology and Dialysis Unit, “Versilia Hospital”, Camaiore, LU Italy; 6 Nephrology and Dialysis Unit, SM Annunziata Hospital, Florence, Italy

**Keywords:** Haemodialysis, Biotechnology

## Abstract

Systemic inflammation and uremic toxins (UT) determine the increased cardiovascular mortality observed in chronic hemodialysis (HD) patients. Among UT, the adipokine Chemerin induces vascular dysfunction by targeting both endothelial and vascular smooth muscular cells (EC and VSMC). As Citrate anion modulates oxidative metabolism, systemic inflammation and vascular function, we evaluated whether citrate-buffered dialysis improves HD efficiency, inflammatory parameters and chemerin-mediated microvascular injury. 45 patients were treated in sequence with acetate, citrate and, again, acetate-buffered dialysis solution (3 months per interval). At study admission and after each treatment switch, we evaluated dialysis efficacy and circulating levels of chemerin and different inflammatory biomarkers. *In vitro*, we stimulated EC and VSMC with patients’ plasma and we investigated the role of chemerin as UT. Citrate dialysis increased HD efficacy and reduced plasma levels of CRP, fibrinogen, IL6 and chemerin. *In vitro*, patients’ plasma induced EC and VSMC dysfunction. These effects were reduced by citrate-buffered solutions and paralleled by the decrease of chemerin levels. Consistently, chemerin receptor knockdown reduced EC and VSMC dysfunction. In conclusion, Switching from acetate to citrate improved dialysis efficacy and inflammatory parameters; *in vitro*, chemerin-induced EC and VSMC injury were decreased by using citrate as dialysis buffer.

## Introduction

Chronic hemodialysis (HD) patients suffer from high cardiovascular morbidity and mortality mainly due to a chronic systemic inflammation coupled with an aberrant metabolic state^1^. Several uremic toxins contribute to HD-related premature vascular senescence: among them, adipokines adversely influence both systemic metabolism and the vascular system. Chemerin is a 14-kDa adipokine up-regulated in HD patients and known to target both the endothelium and vascular muscle cells; moreover, chemerin has been associated with both micro and macro-vascular injury^2,3^.

Acetic acid is widely used as dialysis solution buffer and after a standard HD session its plasmatic levels are several times higher than those observed in physiological conditions^4,5^. Of note, acetic acid has been associated with endothelial dysfunction in several studies: moreover, it represents a privileged substrate for fatty acid synthesis in the liver^4^. Recently, citric acid has been proposed as alternative dialysis buffer due to its anti-coagulant, anti-inflammatory and anti-oxidant properties: indeed, citrate chelates multivalent cations such as iron and copper, restores mitochondrial function, increases glutathione production, reduces complement and neutrophil activation^6,7^. Moreover, since citrate down-regulates the clotting cascade, it also improves dialysis efficiency and reduces endothelial injury^8^.

In this study, a cohort of HD patients was treated in sequence with acetate-, citrate- and then (again) acetate-buffered dialysis solutions to test whether citrate could reduce the systemic inflammation and microvascular dysfunction observed in end stage chronic kidney disease.

## Results

### Study population

Enrolment started on October 30^th^ 2014. Of the 45 enrolled patients, thirty-nine completed the study (by July 2015); 1 dropout was due to diagnosis of invasive cancer, 4 patients underwent kidney transplantation and 1 died during the study. Data from dropout patients were not included in the final analysis. No major adverse events were associated with study protocol. Clinical data at enrollment are summarized in Table 1; main HD related parameters are summarized in Table 2: no significant variations were observed among the different study points (T0: study start, T1 end of the first 3-month acetate period, T2: end of the 3-month citrate period, T3 end of the second 3-month acetate period).

**Table 1:** Main clinical and dialysis-related parameters.

Patients Main Characteristics	
Male/Female	64%/36%
Age (year)	62 + 15
Dialysis vintage (year)	10 + 9
OL-HDF (n)	18% (8)
BIC-HD (n)	82% (37)
**Vascular Access**	
AVF (n)	84% (38)
CVC (n)	16% (7)
Temporary CVC	0 (0%)
**Comorbidities**	
Hypertension	85%
Diabetes	20%
Prev. CAD or Stroke	30%
**Nephrophaty**	
GN	28,7%
DN	19%
ADPKD	20%
Urologic	15,6%
Tubulo-Interstitial	6,7%
Other/Unknown	10%

ADPKD: autosomic dominant polycystic kidney disease; AVF: arteriovenous fistula; BIC-HD: bicarbonate dialysis; CAD: coronary artery disease, CVC: central venous catheter; DN: diabetic nephropathy; GN: glomerulonephritis, OL-HDF: on line hemodiafiltration. Prev.: previous.

**Table 2:** Main dialysis parameters at the different time-points of the study.

Variables	T0		T1		T2		T3		p
	Mean	SD	Mean	SD	Mean	SD	Mean	SD	
Qd	500	0	500	0	500	0	500	0	1
Qb	316,8	25,9	314,2	22,1	311,0	30,4	307,9	21,9	0,65
HD duration	229,2	19,0	230,7	18,6	233,2	19,3	233,6	18,1	0,87
Infusion (OL-HDF only)	22,1	3,3	18,7	3,2	20,5	3,7	23,7	3,5	0,32
UF/session	2,6	0,31	2,5	0,30	2,7	0,32	2,7	0,32	0,78

HD: hemodialysis (any); Qb: blood flow; Qd: dialysis solution flow; OL-HDF: on line hemodiafiltration; SD: standard deviation. UF: ultrafiltration.

### Citrate buffer improved dialysis efficiency

**Figure 1:**
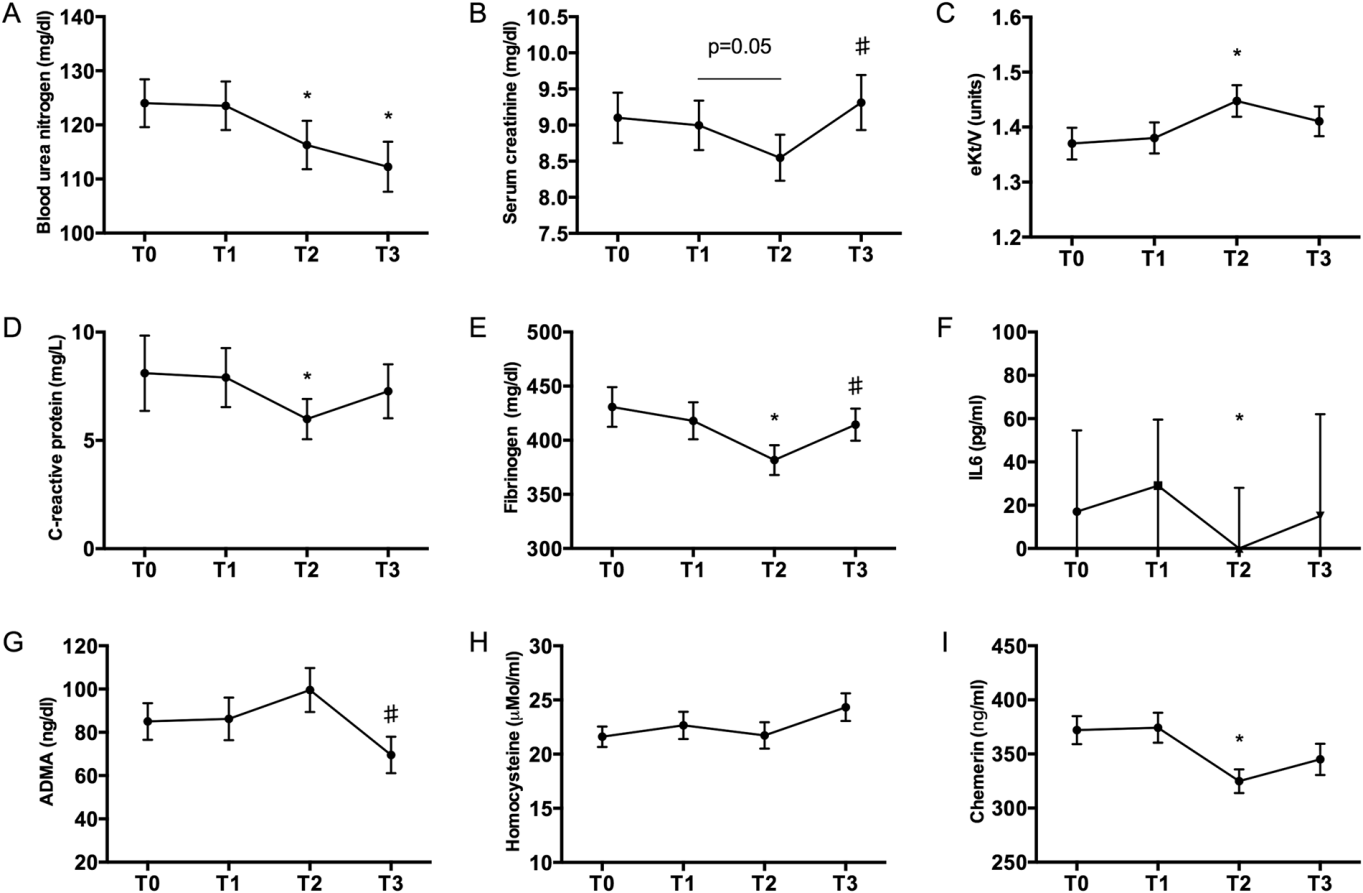
Patients’ clinical data at the different study time points. **(A)** Pre-dialysis blood urea nitrogen levels, **(B)** pre-dialysis serum creatinine values, **(C)** dialysis efficacy estimated with the eKt/V Daugirdas formula **(D)**, pre-dialysis values of plasma C-Reactive Protein (CRP), **(E)** pre-dialysis plasma fibrinogen, **(F)** pre-dialysis serum IL6, **(G)** pre-dialysis serum ADMA values, **(H)** pre-dialysis serum homocystein values and **(I)** pre-dialysis serum Chemerin measured at the different study time-points. T0: study start; T1: end of 1^st^ acetate period (3 months from study start); T2 end of citrate (6 months); T3 end of 2^nd^ acetate period (9 months). *p < 0.05 when data were compared with T1; ^♯^p < 0.05 when data were compared with T2.

Dialysis efficacy increased after treatment with citrate buffer and subsequently worsened when acetate-containing solution was used (Fig. 1A–C); for all studied parameters, no significant variations were observed between T0 and T1 or T1 and T3. The decrease in pre-dialysis serum creatinine from T1 to T2 was not significant (9,0 ± 2,1vs. 8,5 ± 1,9 mg/dl; p = 0,05), whereas the increase between T2 and T3 was significant (8,5 ± 1,9 vs. 9,4 ± 2,4; p = 0,003). Pre-HD urea was significantly reduced between T1 and T2 (p = 0,04), and almost unchanged between T2 and T3, no changes were observed in post-dialysis urea levels (Supplementary Fig. 1). Dialysis efficacy was assessed by urea kinetic model according to Daugirdas’ equation^9^; eKt/V significantly increased from T1 to T2 (1,38 ± 0,17 vs.1,45 ± 0,18; p = 0,01). Data were also analyzed according to dialysis type (standard bicarbonate HD n = 31 vs. HDF n = 8 – Supplementary Figs 2 and 3).

### Citrate dialysis improved parameters of systemic inflammation

Inflammation-related biomarkers decreased from T1 to T2 and subsequently worsened at T3; not all variations reached statistical significance (Fig. 1D–I and Supplementary Figs 2 and 3). In all analysis, no significant differences were observed between T0 and T1 or T1 and T3. CRP varied from 7,9 ± 8,5 mg/dl at T1 to 5,9 ± 5,7 at T2 (p = 0,031). Fibrinogen decreased from 417 ± 106 mg/dl at T1 to 381 ± 85 at T2 and was 414 ± 92 at T3 (T1 vs. T2 p = 0,01; T2 vs. T3 p = 0,006). No significant homocystein variations were observed; surprisingly, ADMA increased from 86 ± 61 ng/ml at T1 to 99 ± 63 at T2 and then decreased to 69 ± 52 at T3 (T1 vs. T2 p = 0,39; T2 vs. T3 0,01). Finally, the adipokine chemerin decreased from T1 to T2, this variation was significant also when patients were analyzed according to dialysis type (standard bicarbonate HD vs. HDF – Supplementary Figs 2 and 3). No significant differences of calcium-phosphorus, β2-microglobulin, hemoglobin, erythropoietin resistance index (ERI), acid-base status, adverse events (i.e. hypotension, hypoglycemia, clotting etc.), dialysis solution sodium and potassium or OL-HDF reinfusion volume were observed (data not shown). As Chemerin has been related to patient body weight and nutritional status^10^, we evaluated post-dialysis body weight and normalized protein catabolic rate (nPCR, a validated index of dialysis patients’ nutrition state) at all study time points (Supplementary Fig. 4). No changes were observed in these 2 parameters.

### *In vitro* studies on human endothelial cells (EC) and vascular smooth muscle cells (VSMC)

Uremic *milieu* is known to cause microvascular dysfunction trough several mechanisms including a direct cytotoxic effect of uremic toxins on EC and VSMC^11,12^. Hence, we investigated *in vitro* whether the shift from acetate to citrate buffer may improve cell function: to test this hypothesis, we performed functional assays on EC and VSMC using patients’ plasma collected at different time points.

**Figure 2:**
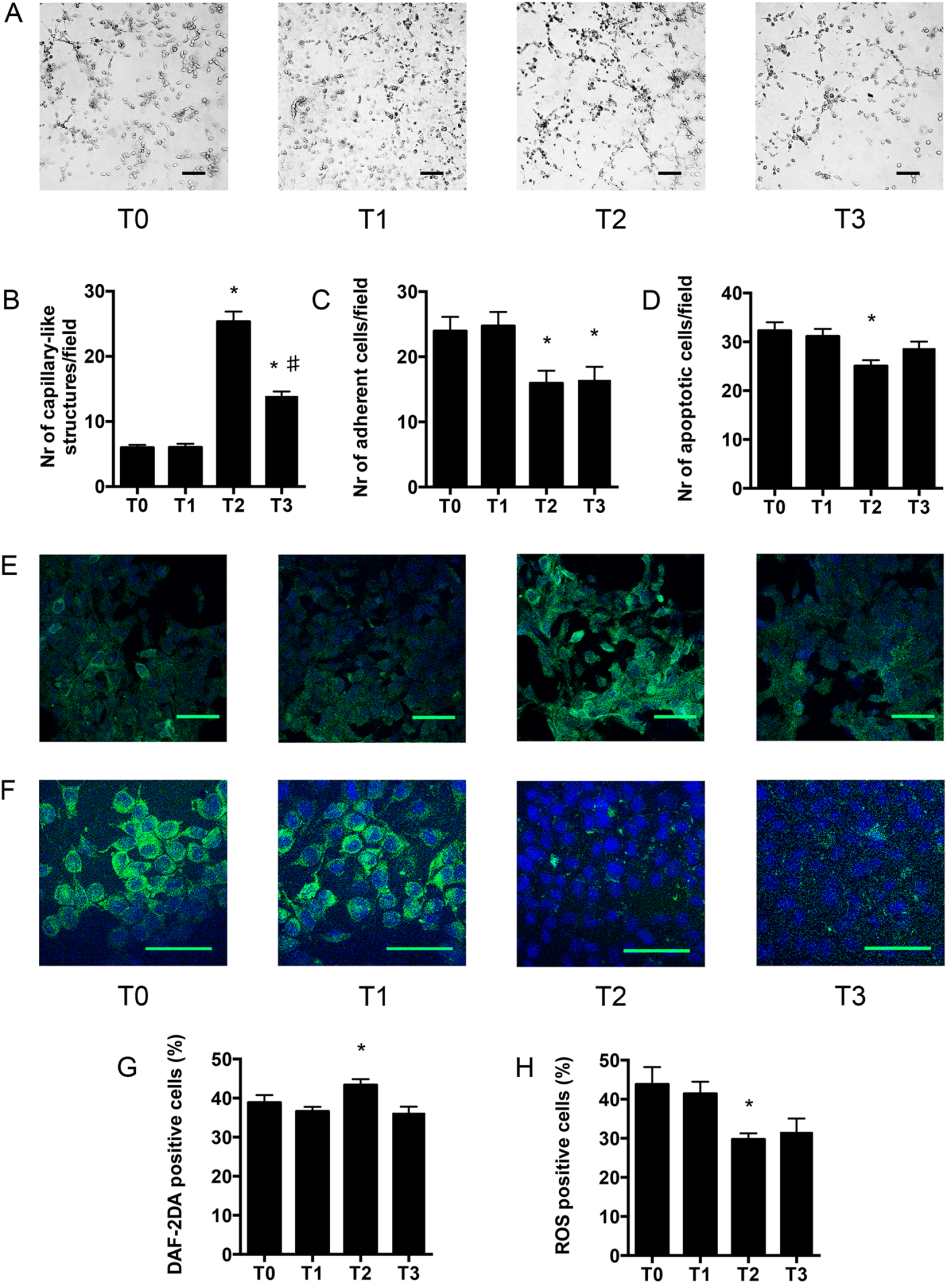
Effect of Citrate buffered-dialysis on human endothelial cells (EC). In all the experiments EC were incubated with patients’ plasma collected at different study time points. **(A)** Representative micrographs of EC angiogenesis on matrigel. **(B)** Quantification of the capillary-like structures generated by EC on matrigel-coated plates. (**C)** Number of adherent Peripheral Blood Mononuclear Cells (PBMC) on a layer of confluent EC. **(D)** % of EC apoptosis (TUNEL test). **(E)** Representative immunofluorescence micrographs of DAF2DA probe staining in EC. **(F)** Representative immunofluorescence micrographs of ROS probe staining in EC. **(G)** Flow cytometry (FACS) quantification of Nitric Oxygen (NO) bioavailability by DAF2A probe staining. **(H)** Flow cytometry (FACS) Quantification of Reactive Oxygen Species (ROS) generation in EC by appropriate fluorescent probe. T0: serum from study start; T1: end of 1^st^ acetate period (3 months from study start); T2 end of citrate treatment (6 months); T3 end of 2^nd^ acetate period (9 months). *p < 0.05 when data were compared with T1; ^♯^p < 0.05 when data were compared with T2. Scale bar length is 50 μm in all micrographs.

In presence of T2 plasma EC angiogenesis on Matrigel was improved (Fig. 2A,B) and a decreased adhesion of fluorescent peripheral blood mononuclear cells (PBMC) on EC was observed (Fig. 2C). Consistently, EC apoptotic rate was significantly decreased after culture with T2 plasma, when compared with plasma from T0, T1 and T3 (Fig. 2D).

To test whether the decrease in EC apoptosis and angiogenesis *in vitro* were associated with an improved cell function, we investigated endothelial NO and ROS production by using dedicated fluorescent assays (Fig. 2E–H). Compared to T0-1 or T3, NO production was improved by the use of T2 plasma. Of interest, the increase in NO activity was not related to ROS over-production; conversely, ROS were down regulated in cells stimulated with T2 plasma.

**Figure 3:**
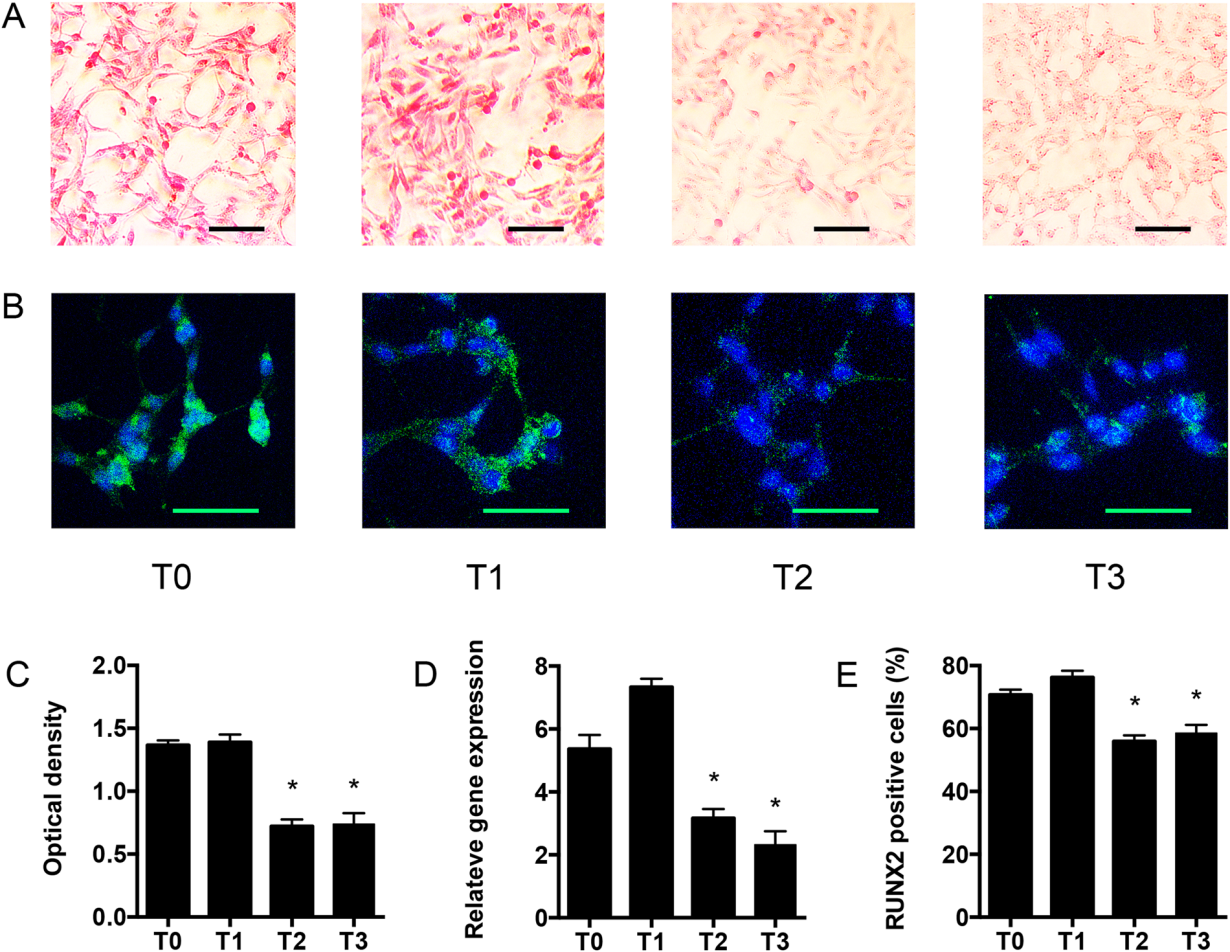
Effect of Citrate buffered-dialysis on human vascular smooth muscle cell (VSMC). In all the experiments VSMC were incubated with patients’ plasma collected at different study time points. **(A)** Representative micrographs of red alizarin staining of VSMC. **(B)** Representative immunofluorescence micrographs of RUNX2 staining of VSMC. **(C)** Spectrophotometry quantification of red alizarin internalization by VSMC. **(D)** RUNX-2 mRNA relative quantification by qRT-PCR (quantitative Reverse Transcription Polymerase Chain Reaction) in VSMC. **(E)** Flow cytometry (FACS) quantification of RUNX-2 positive VSMC. T0: serum from study start; T1: end of 1^st^ acetate period (3 months from study start); T2 end of citrate treatment (6 months); T3 end of 2^nd^ acetate period (9 months); O.D.; optical density. *p < 0.05 when data were compared with T1; ^♯^p < 0.05 when data were compared with T2. Scale bar length is 50 μm in all micrographs.

Alizarin red staining was used to detect intracellular calcification of VSMC (Fig. 3); intracellular calcium deposits were significantly reduced when cells were stimulated with plasma collected after the citrate dialysis period (T2), and this beneficial effect persisted after citrate discontinuation. In order to confirm these results, we evaluated by immunofluorescence, qRT-PCR and FACS the expression of RUNX2, a transcription factor strongly associated with VSMC calcification^13^. We found that RUNX2 expression was reduced after stimulation with T2 plasma in respect to T0, T1 or T3 plasma.

### Inhibition of chemerin signaling by specific small interfering RNA (siRNA) in EC and VSMC

**Figure 4:**
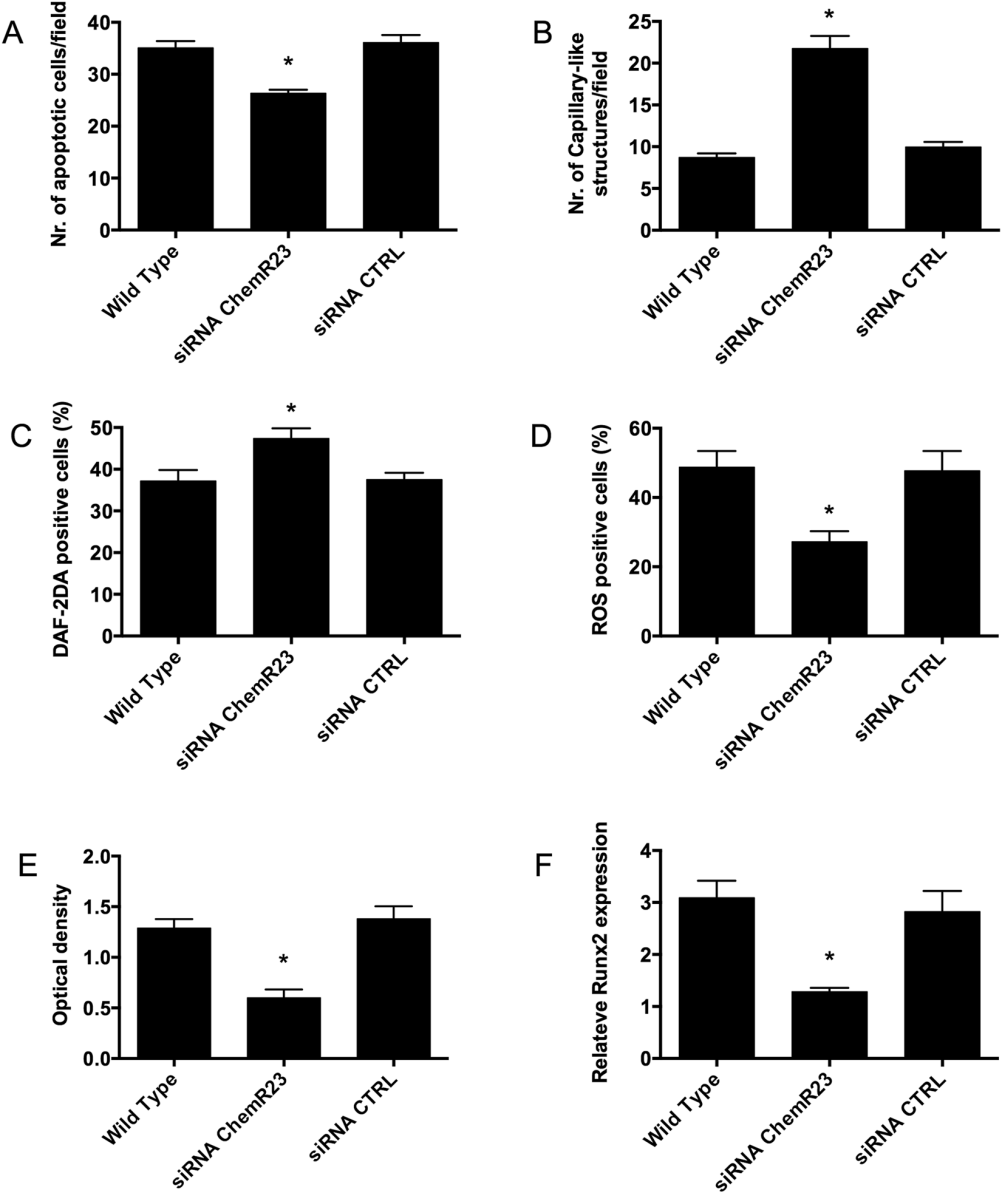
Effect of Chemerin knockdown on human endothelial and Vascular Smooth Muscle Cell (EC and VSMC). Human EC and VSMC were stimulated with the plasma from patients showing the highest chemerin levels at study start (n = 5). The following experiments were performed in non-engineered wild type cells (WT), after silencing of Chemerin receptor (siRNA ChemR23) or with an irrelevant control siRNA (siRNA CTRL). **(A**) Quantification of apoptotic EC number (TUNEL assay). **(B)** Quantification of capillary-like structures (angiogenesis assay) generated by EC on Matrigel-coated plates. **(C)** Flow cytometry (FACS) Quantification of Nitric Oxygen (NO) bioavailability by DAF2A probe staining in EC. **(D)** Flow cytometry (FACS) Quantification of Reactive Oxygen Species (ROS) generation in EC by appropriate fluorescent probe. **(E)** Spectrophotometry quantification of red alizarin internalization by VSMC. **(F)** RUNX2 mRNA expression by qRT-PCR (quantitative Reverse Transcription Polymerase Chain Reaction). *p < 0.05 siRNA ChemR23 vs. wild type or siRNA control.

According to our data, citrate dialysis improved a number of inflammatory parameters potentially affecting vascular function. To specifically evaluate the role of chemerin as mediator of vascular injury, we engineered EC and VSMC with an appropriate siRNA to knockdown the chemerin receptor ChemR23 (Supplementary Fig. 5). Knockdown and control cells were stimulated with T0 samples from the 5 patients with the highest chemerin plasma levels (Fig. 4). After ChemR23-knockdown, EC displayed reduced apoptosis and ROS production, increased NO release and angiogenesis when compared to cells transfected with irrelevant-siRNA or wild type cells. Additionally, VSMC transfected with ChemR23-siRNA showed decreased alizarin-red staining and reduced RUNX2 mRNA expression.

## Discussion

In this study, the use of citrate buffer was associated with enhanced dialysis efficacy and a reduction of systemic inflammation parameters in a cohort of 45 hemodialysis patients followed for 9 months. We observed a reduction of pre-dialysis creatinine and urea levels, an improvement of eKt/V and a decrease in several inflammation biomarkers such as CRP, fibrinogen, IL6 and the adipokine chemerin. To validate our clinical findings and the potential role of chemerin in microvascular dysfunction, we investigated *in vitro* the effects of patients’ plasma on cultured EC and VSMC. For both cell types, the cytotoxicity induced by uremic plasma was partially reverted when citrate-dialysis serum was tested. Finally, we observed that chemerin is a key mediator of vascular cell injury: indeed, the knockdown of its specific receptor ChemR23 on EC and VSMC surface associated with an improved cell function.

Despite several technical advances in the extracorporeal techniques, the high mortality rate associated with HD is still far to be controlled. Several studies demonstrated that young and non-comorbid HD patients have a cardiovascular senescence comparable to elderly subjects without renal failure^14–16^; on the other hand, the association of dialysis need and other major comorbidities exposes patients to an extremely high cardiovascular risk^1,17,18^. For instance, diabetic patients with end stage renal disease have a 2-year mortality close to 50%^19,20^. Fluid overload and electrolyte disorders are major determinants of cardiovascular risk but do not explain the whole mortality excess observed in HD patients. Uremic toxin accumulation is responsible for direct cytotoxicity on large vessels and microvascular networks^11,12,14^. Indeed, smooth muscle calcification and chronic endothelial dysfunction are two main causes of microvascular injury and are sustained by several molecules overexpressed in the uremic *milieu*.

Citrate anion is the cornerstone of eukaryotic oxidative metabolism trough the citric acid cycle: moreover, it chelates and shuttles several polyvalent cations, (i.e. calcium, zinc, silver, copper etc.) and contributes to acid-base balance^6^. Citrate is also a key regulator of mineral bone metabolism as it stabilizes hydroxyapatite crystals and reduces bone reabsorption^21^. In common clinical practice, citrate is effective in treatment of CKD-related metabolic acidosis, reduces urinary tract inflammation and inhibits calcium phosphate, calcium oxalate and uric acid nephrolithiasis. In the last decade, the use of citrate in extracorporeal renal replacement therapy for Acute Kidney Injury (AKI) lead to an authentic revolution: citrate-based local anticoagulation is more effective than other strategies (i.e. heparin and direct thrombin inhibitors) and is almost free from systemic adverse effects^22^. Additionally, some authors found that regional citrate anticoagulation down-regulates complement activation, neutrophil degranulation and IL1-β secretion^23,24^. Subsequently, citrate has been proposed as dialysis solution buffer in chronic HD; indeed, preliminary studies demonstrated an intra-dialytic hemodynamic improvement and a reduced use of heparin^6,7^.

The impact of citrate on dialysis efficacy is controversial; in a recent study on 44 patients treated with on-line hemodiafiltration (ol-HDF) citrate-buffer did not improve treatment efficacy^25^. However, other authors have reached different conclusions: Kossmann *et al*.^26^ found a significant increase in Kt/V in 142 patients treated with citrate-buffered hemodialysis (non HDF) for 6 months. In accordance with Kossmann’s study and with the results of the present study, other groups have found an increased dialyzer half-life^27^ and a stable dialyzer clearance despite a 30% heparin reduction^28^ in patients treated with citrate-buffered dialysis. Our study enrolled 45 patients, of which only 8 were treated with ol-HDF; interestingly, we could not observe the improvement of eKt/V in this sub-group. Thus, one could speculate that, due to the higher clearances observed in ol-HDF treatments, a large number of patients is required to highlight a possible citrate effect.

The observed increase in dialysis efficacy may be partially explained by the anticlotting proprieties of citrate. A reduction in circuit clotting was not detected, probably as a consequence of the few episodes observed (n = 4); however, it is reasonable to hypothesize that the down-regulation of the coagulation cascade contributed to maintain the efficiency of the dialyzer. Moreover, reduced fibrinogen levels may decrease also blood viscosity and the formation of the so-called “protein cake” (the layer of proteins that progressively coats the dialyzer membrane, thus affecting treatment efficiency)^29^.

Adipokines are a family of soluble proteins produced by fat tissue that act as endocrine and paracrine factors. These proteins regulate lipid metabolism, the sense of hunger and satiety and many endocrine processes including insulin secretion. Dysregulation of adipokine signaling has been recently associated with obesity, diabetes, dyslipidemia and chronic inflammation^30,31^. In the last years, an increasing body of evidence pointed out the role of adipokines as uremic toxins and inflammatory mediators; among adipokines, chemerin has been related to CKD progression and leucocyte recruitment in renal tissues^32,33^. In recent studies, chemerin was also found to positively correlate with systemic inflammation biomakers (i.e. CRP, WBC count), insulin resistance, dyslipidemia and hepatic disorders in incident and maintenance HD patients^34,35^.

In this study, citrate dialysis reduced circulating chemerin levels together with IL6, CRP and fibrinogen. As citrate improves mitochondrial function and oxidative metabolism^36^, one could speculate that it may directly inhibit chemerin expression or signaling. Of interest, the improved chemerin levels persisted after the suspension of citrate-buffered dialysis and some of the beneficial effects observed *in vitro* by using T2 plasma were maintained also by using T3 plasma samples.

Nitric Oxide (NO) is known to improve endothelial tropism and function by activating adenylate-ciclase intracellular pathway; however, in oxidant conditions, NO release may increase Reactive Oxygen Species (ROS), thus triggering tissue injury. In our *in vitro* experiments, citrate HD (T2) plasma improved the activity of the NO-synthase in EC, but this effect was associated to a reduced ROS production. Moreover, T2-plasma stimulated EC were less prone to leucocyte recruitment as a probable consequence of the weaker expression of surface adhesion molecules.

Smooth muscle calcification is another pathognomonic feature of uremic vascular dysfunction and leads to tunica media mineralization, a lesion known as Monckeberg arterial sclerosis. Our data confirmed that uremic plasma induces myocyte trans-differentiation and intracellular calcification by promoting the osteoblastic transcription factor RUNX2^13,37^. Also in this case, citrate use was associated with a reduced cell dysfunction characterized by decreased calcium deposits (stained by alizarin red) and down-regulation of RUNX2 mRNA.

To further investigate whether chemerin is a possible mediator of the observed cytotoxic effects, we selected the 5 patients with the highest circulating levels of this adipokine (all at T0) and we repeated the same *in vitro* assays after chemerin-receptor (ChemR23) knockdown by specific siRNA. Our data demonstrated that chemerin inhibition exerted a protective effect (on both EC and VSMC) similar to what observed in presence of plasma from citrate HD. Interestingly, our data are consistent with a previous investigation reporting an enhanced production of ROS in endothelial cells stimulated by chemerin; consistently with our results, this finding was correlated with an impaired eNOS activity^2^.

Our data suggest that citrate-buffered solutions may modulate several clinical and biochemical parameters of HD patients: however, the present study presents some limits. For a number of studied parameters we found a significant improvement from T1 to T2 but not a corresponding worsening after citrate discontinuation (T2 vs. T3), this might be related to persisting beneficial effects of citrate treatment but further studies are needed to untangle these findings. As this study is mono-centric, larger investigations are needed to confirm our results. Moreover, our patients had a relatively low prevalence of diabetes when compared to large HD registry studies^38,39^, thus our data might be not fully applicable to other patients cohorts. Finally, our therapeutic intervention had two components (i.e.: acetate removal and citrate use) and we cannot discern their relative contribution to the observed improvements.

In conclusion, the switch from acetate to citrate buffer increased dialysis efficacy and simultaneously decreased chronic inflammation parameters. Our *in vitro* data demonstrated that citrate-dialysis prevents EC dysfunction and VSMC osteoblastic differentiation, identifying the adipokine chemerin as a possible target to inhibit microvascular injury.

## Patients and Methods

### Inclusion and exclusion criteria

In this monocentric study we enrolled 45 patients from Città della Salute e della Scienza University Hospital, Torino, Italy. Inclusion criteria were: age >18 years, creatinine clearance <5 ml/min, treatment with bicarbonate HD (BIC-HD) or on-line hemodiafiltration (OL-HDF) 3 times per week for at least 6 months. Patients with neoplastic, acute or chronic inflammatory diseases (including active HIV, HCV or HBV infection) were excluded. A written informed consent was obtained and the study was conducted in accordance with the declaration of Helsinki. Study protocol was reviewed and approved by local ethic committee (Comitato Etico Interaziendale A.O.U. Città della Salute e della Scienza di Torino - A.O. Ordine Mauriziano - A.S.L. Città di Torino; study registration number nr. ^2^CEI 753 – 0064476, June 2013). The trial was also registered on www.clinicaltrial.gov (ID: NCT03577249, registration date: June 13, 2018).

### Treatment protocol and collected parameters

Total study duration was 9 months; in the first 3 months patients were treated with a standard dialysis solution containing 3 mmol/l acetate (Select Bag, Baxter Gambro Renal, New Providence NJ), the following 3 months we used an acetate-free solution containing 1 mmol/L citrate (Select Bag Citrate) and the last 3 months again the acetate solution. Except for citrate/acetate content the 2 solutions were identical; sodium and potassium concentration varied according to patient needs. Each patient maintained the same dialysis modality for all the study length (BIC-HD or OL-HDF). We collected patients’ plasma at study start (T0) and at the end of the 3 treatment intervals (T1, 2 and 3). Samples were used to perform *in vitro* experiments and to measure inflammatory markers (CRP, fibrinogen, IL6, homocystein, chemerin, and asymmetric di-methyl-arginine – ADMA), markers of dialysis efficiency (creatinine, urea, β2-microglobulin) and other parameters related to end stage kidney disease (calcium, phosphorus, parathyroid hormone, acid-base status, hemoglobin). Basing on recorded variables, we estimated erythropoietin resistance index (ERI) and dialysis efficacy (eKt/V using Daugirdas formula). During each dialysis session, patients were monitored according to the best clinical practice of the center^17^, intradialytic adverse events (i.e. hypotension, hypertension peak, dialysis lines clotting, arrhythmias) were recorded. To account for systemic recirculation, post-dialysis blood sampling was obtained from the line after have paused dialysis for 5 minutes. Patients were screened for vascular access recirculation by glucose infusion test^40^ when clinically indicated or routinely at least once a month.

### Study sample estimation and statistical analysis

The main outcome variable considered for sample size estimation was CRP. Assuming that citrate buffered dialysis would have reduced CRP by 30% over 3 months, we calculated that the inclusion of 34 patients would have conferred an 80% power to demonstrate a statistically significant reduction (α = 0.05, one-tailed test) of this parameter. To account for a 30% drop-out rate, we included in the study a total of 45 subjects.

Data analysis was performed by Prism (GraphPad, La Jolla, CA). All data were subjected to D’Agostino-Pearsons test to assess variables distribution; continuous non-normal distributed variables were analyzed with Mann-Whitney test; normal distributed variables were analyzed with two-tails paired t-test or one way ANOVA and Bonferroni multi-comparison test. A p value <0.05 was considered statistically significant. In all figures, clinical data are depicted as mean ± standard deviation (SD) or median ± interquartile range (IQR) where appropriate, *in vitro* data are depicted as mean ± standard error mean (SEM).

### *In vitro* studies

*In vitro* methods are described in details in Supplementary Information.

## Supplementary information

Supplementary information

## Data Availability

The datasets generated during and/or analysed during the current study are available from the corresponding author on reasonable request.
