# Supplementary material for: Citrate anion improves chronic dialysis efficacy, reduces systemic inflammation and prevents Chemerin-mediated microvascular injury
Source: Sci Rep. 2019 Jul 23;9:10622. doi: 10.1038/s41598-019-47040-8 (PMC6650610; doi:10.1038/s41598-019-47040-8)
Supplement: Supplementary file 1 — Supplementary information [file 41598_2019_47040_MOESM1_ESM.pdf]

Citrate anion improves chronic dialysis efficacy, reduces systemic inflammation and prevents Chemerin-mediated microvascular injury

Supplementary information

Sergio Dellepiane[1*], Davide Medica[1*], Cesare Guarena[1], Tiziana Musso[2], Alessandro Domenico Quercia[3], Gianluca Leonardi[1], Marita Marengo[4], Massimiliano Migliori[5], Vincenzo Panichi[5], Luigi Biancone[1], Francesco Pizzarelli[6], Giovanni Camussi[1], Vincenzo Cantaluppi[3]

[1]Nephrology, Dialysis and Kidney Transplantation Unit, Department of Medical Sciences and [2]Microbiology and Virology Unit, Department of Pathology, University of Torino, "Città della Salute e della Scienza" University Hospital, Torino, Italy;

[3]Nephrology and Kidney Transplantation Unit, Department of Translational Medicine and Center for Autoimmune and Allergic Diseases (CAAD), University of Piemonte Orientale (UPO), "Maggiore della Carità" University Hospital, Novara, Italy;

[4]Nephrology and Dialysis Unit, ASLCN1, Cuneo, Italy;

[5]Nephrology and Dialysis Unit, "Versilia Hospital", Camaiore (LU), Italy.

[6]Nephrology and Dialysis Unit, SM Annunziata Hospital, Florence, Italy.

*SD and DM equally contributed to the paper

Supplementary methods: in vitro studies

Cell isolation and culture

Human umbilical vein-derived endothelial cells (EC) were obtained by ATCC (PCS-100- 010-ATCC, Manassas VA). EC were plated with EBM medium supplemented with 10% fetal calf serum (FCS – GE Health Care, Boston MA) and different endothelial growth factors (Lonza, Basel, Switzerland). Human Vascular Smooth Muscular Cells (VSMC) were obtained by ATCC and grown in Dulbecco Modified Eagle Medium (DMEM – GE Health Care) with 10% of FCS. Experiments were performed without FCS and after 24h (EC except angiogenesis), 72h (angiogenesis assay) or 96h (VSMC) incubation with patients' plasma diluted 1:10. Peripheral Blood Mononuclear Cells (PBMC) were obtained from healthy volunteer after isolation with Ficoll-Hypaque (GE Health Care) density gradient and added to cell cultures in selected experiments.

Functional assays

Cell death: EC were cultured in 96-well plates, incubated with appropriate stimuli, subjected to TUNEL assay (Chemicon Int. Temecula, CA) and analyzed under a fluorescence microscope to detect stained cells in 10 non-consecutive fields.

Nitric Oxide and Reactive Oxygen Species production: Nitric Oxide Synthase (NOS) activity was assessed in EC by 4,5-diaminofluorescein diacetate assay (DAF-2 DA – Enzo Life Sciences, Inc, Farmingdale, NY). DAF-2 DA is a precursor of the fluorescent dye triazolofluorescein that is activated by NOS. Reactive Oxygen Species (ROS) production was assessed by Image–iT® Detection Kit (Life Technologies, Carlsbad, CA, USA). The assay is based on 5-(and-6)-carboxy-2′,7′-dichlorodihydrofluorescein diacetate (carboxyH2 DCFDA), a compound that releases fluorescence after binding ROS. Experiments were conducted according to manufacturers' instructions. After incubation with selected stimuli, cells were analyzed under a fluorescence microscope or re-suspended with EDTA and analyzed by FACS.

Cell adhesion: PBMC were labeled for 15 minutes with 10 μm of fluorescent Vybrant Cell Tracer kit (Life Technologies) in RPMI. Fluorescent cells were re-suspended in EBM without FCS (50×10^6/ml) and added to confluent monolayer of EC on six-well plates. After 1hr at 37°C of slight agitation, samples were washed, fixed with 4% paraformaldehyde and observed by UV light microscopy. Fluorescent cells were quantified in 10 different fields at x200 magnification.

Angiogenesis on Matrigel: EC were cultured on growth factor reduced Matrigel (Becton Dickinson, San Jose, CA) in EBM enriched by selected stimuli. After 12h, EC were observed under a Nikon-inverted microscope (Nikon, Kanagawa, Japan). Image analysis was performed with the MicroImage analysis system (Casti Imaging, Venice, Italy).

Cell calcification: intracellular calcium deposits were quantified by red-alizarin staining. VSMC were cultured in 24-well plates and fixed with 4% paraformaldehyde in PBS for 45 min at 4°C. Cells were then washed in distilled water, exposed to Alizarin Red (2% aqueous, Sigma Aldrich, St Louis, MO) for 5 min, washed again and analyzed by inverted light microscopy. Cell lysates were analyzed in an automatized spectrophotometer at a wavelength of 570 nm.

Quantitative RT-PCR for RUNX2: total RNA was extracted by VSMC after appropriate stimulation using mirVana RNA isolation kit (Life Technologies). RNA concentration and purity were detected by the NanoDrop1000 spectrophotometer. We evaluated RUNX2 mRNA expression by using High cDNA Reverse Transcription Kit (Applied Biosystems, Foster City, CA, USA) and the Power SYBR Green PCR Master Mix on a 96-well StepOnePlus Real Time System (Applied Biosystems). Actin-β was used as housekeeping gene. Fold change in RNA expression was calculated as $2^{-\Delta\Delta Ct}$ using the geometric mean in Ct values as normalizer. The following primers were used:

- Actin-β: forward, 5’-GAG TCC GGC CCC TCC AT-3’; reverse; 5’-GCA ACT AAG TCA TAG TCC GCC TAG A-3’
- RUNX2: forward 5'- GGA GTG GAC GAG GCA AGA GTT -3'; reverse 5'- CTG TCT GTG CCT TCT GGG TTC -3';

Analysis of RUNX2 protein expression in VSMC: For immunofluorescence, VSMC cultured on chamber slides (Thermo Scientific, Waltham, MA, USA) were fixed in ethanol/acetic acid 2:1 and incubated with primary rabbit polyclonal antibodies directed to human RUNX2 (Santa Cruz Biotechnology, Santa Cruz, CA, USA). Slides were washed with PBS and then incubated with Alexa Fluor–conjugated secondary antibodies (Life Technologies) for 30 min at 4°C. All samples were counterstained by 2.5 μg/ml Hoechst (Sigma Aldrich) for 5 minutes, mounted with anti-fade mounting medium (Sigma Aldrich), and examined by confocal microscopy (LSM5 PASCAL; Zeiss, Jena, Germany). For FACS analysis, cells cultured in 12-well plates were detached with EDTA and stained for 30m at 4°C with primary antibodies directed to human RUNX2 (Santa Cruz Biotechnologies). After washing, VSMC were incubated with FITC–conjugated secondary antibodies (Sigma Aldrich) for 30 min at 4°C. All incubation periods were performed using a medium containing 0.25% BSA and 0.0016% sodium azide (Sigma Aldrich). Finally, cells were

newly washed, fixed in 4% paraformaldehyde, and analyzed by FACS (Becton Dickinson, Mountain View, CA).

RNA interference: in selected experiments, EC and VSMC were seeded on 6-well plates and engineered to knockdown ChemR23, the chemerin receptor, by transfection with 80 pM of specific siRNA (incubation time: 72h). Transfection with 80 pM of irrelevant siRNA was used as experimental control (all reagents by Santa Cruz Biotechnology). The knockdown of ChemR23 was verified by IF and FACS analysis with an appropriate Ab (Santa Cruz Biotechnology) (Supplementary Figure 5).

Supplementary Figures and Legends

Supplementary Figure 1

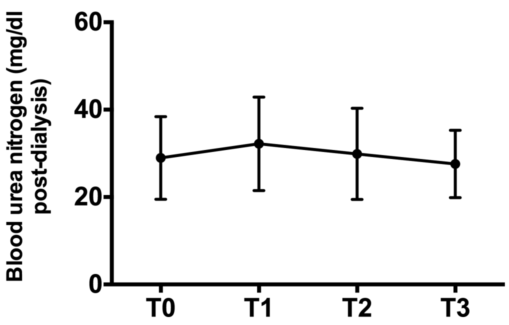

Supplementary Figure 1: Post-dialysis Urea. Post-dialysis blood urea nitrogen levels, at the different study time-points. T0: study start; T1: end of 1st acetate period (3 months); T2 end of citrate (6 months); T3 end of 2nd acetate period (9 months).

Supplementary Figure 2

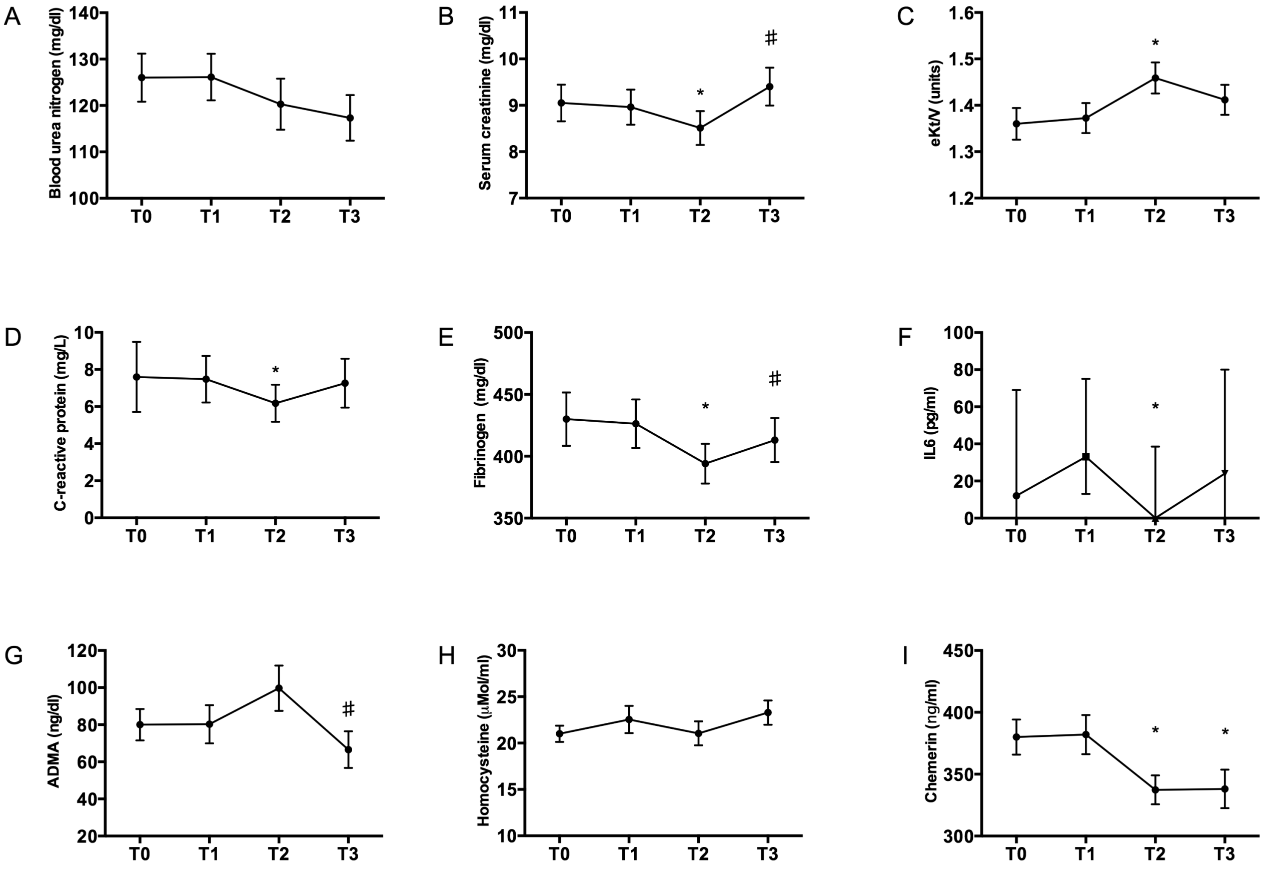

Supplementary Figure 2: Patients' clinical data at the different study time points – standard bicarbonate hemodialysis group (BIC-HD, n=31). (A) Pre-dialysis blood urea nitrogen levels, **(B)** pre-dialysis serum creatinine values, **(C)** dialysis efficacy estimated with the eKt/v Daugirdas formula **(D)**, pre-dialysis values of plasma C-Reactive Protein (CRP), **(E)** pre-dialysis plasma fibrinogen, **(F)** pre-dialysis serum IL6, **(G)** pre-dialysis serum ADMA values, **(H)** pre-dialysis serum homocystein values and **(I)** pre-dialysis serum Chemerin measured at the different study time-points. T0: study start; T1: end of 1st acetate period (3 months); T2 end of citrate (6 months); T3 end of 2nd acetate period (9 months). *: $p<0.05$ when data were compared with T1; ♯: $p<0.05$ when data were compared with T2.

Supplementary Figure 3

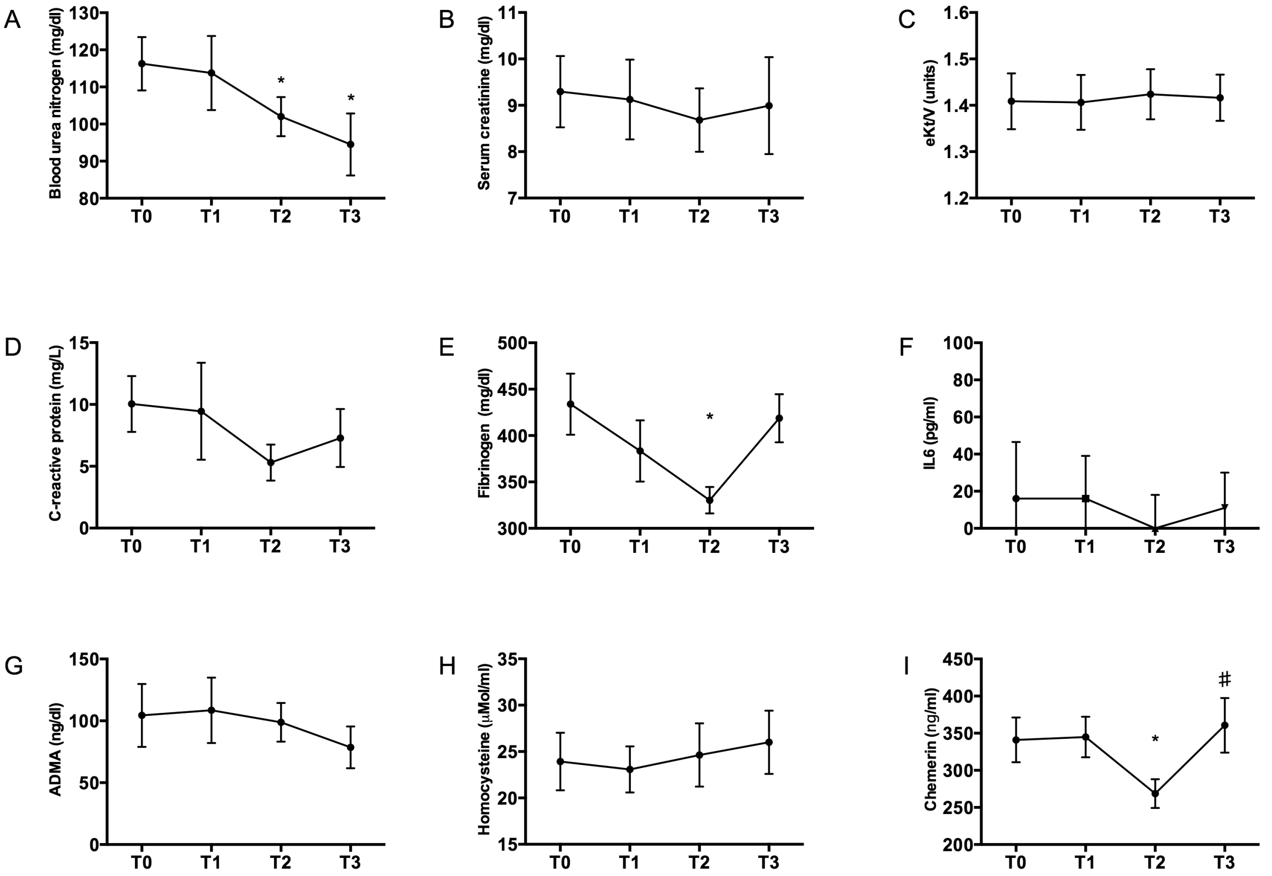

Supplementary Figure 3: Patients' clinical data at the different study time points – online hemodiafiltration group (ol-HDF, n=8). (A) Pre-dialysis blood urea nitrogen levels, **(B)** pre-dialysis serum creatinine values, **(C)** dialysis efficacy estimated with the eKt/v Daugirdas formula **(D)**, pre-dialysis values of plasma C-Reactive Protein (CRP), **(E)** pre-dialysis plasma fibrinogen, **(F)** pre-dialysis serum IL6, **(G)** pre-dialysis serum ADMA values, **(H)** pre-dialysis serum homocystein values and **(I)** pre-dialysis serum Chemerin measured at the different study time-points. T0: study start; T1: end of 1st acetate period (3 months); T2 end of citrate (6 months); T3 end of 2nd acetate period (9 months). *: $p<0.05$ when data were compared with T1; ♯: $p<0.05$ when data were compared with T2.

Supplementary Figure 4

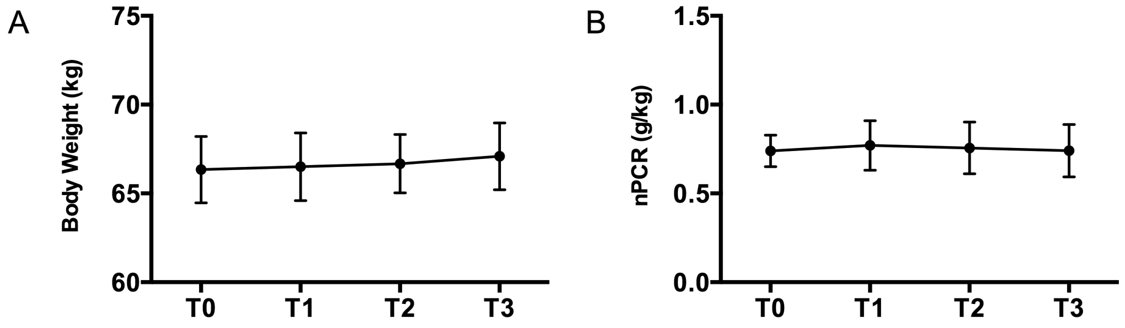

Supplementary Figure 4: Patients' weight and normalized protein catabolic rate (nPCR). (A) Post-dialysis body weight. **(B)** Normalized protein catabolic rate (nPCR). T0: study start; T1: end of 1st acetate period (3 months); T2 end of citrate (6 months); T3 end of 2nd acetate period (9 months). *: $p<0.05$ when data were compared with T1; ♯: $p<0.05$ when data were compared with T2.

Supplementary Figure 5

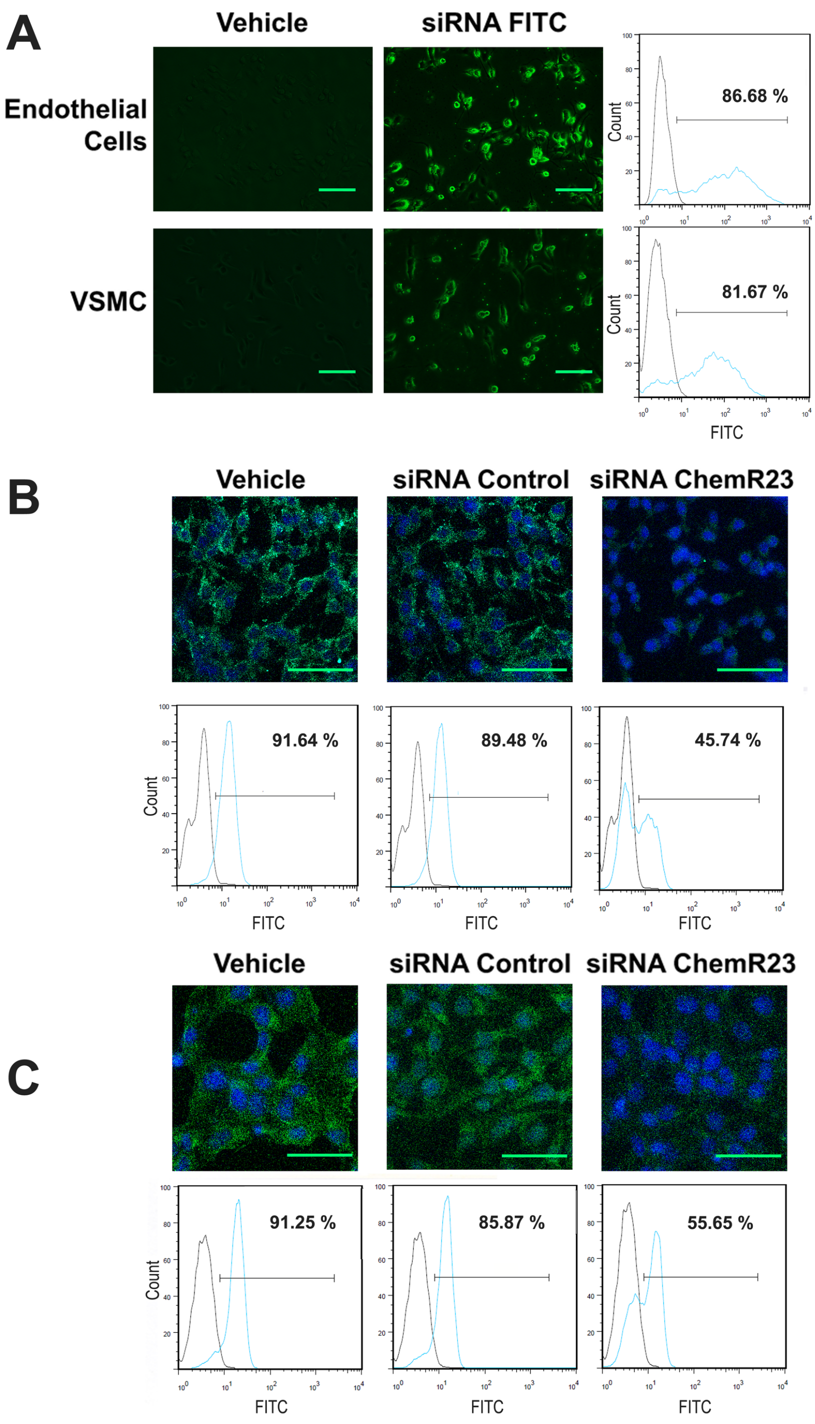

Supplementary Figure 5: ChemR23 small interfering RNA (siRNA) in endothelial cells (EC) and vascular smooth muscular cells (VSMC). (A) Representative immunofluorescence micrographs and FACS quantification of EC and VSMC transfection with a FITC-conjugated siRNA. The experiments show the efficiency of siRNA internalization. **(B)** Representative immunofluorescence micrographs and FACS-quantification of ChemR23 expression (FITC) in EC

after 72h incubation with normal cell-medium, an irrelevant or ChemR23 siRNA, respectively. Nuclei were counter-stained by Hoechst (blue). **(C)** Representative immunofluorescence micrographs and FACS quantification of ChemR23 expression in VSMC after 72h incubation with normal cell-medium, an irrelevant or ChemR23 siRNA, respectively. Nuclei were counter-stained by Hoechst (blue). Scale bar length is 50 µm in all micrographs.
